# Biogenic Hierarchical TiO_2_/SiO_2_ Derived from Rice Husk and Enhanced Photocatalytic Properties for Dye Degradation

**DOI:** 10.1371/journal.pone.0024788

**Published:** 2011-09-09

**Authors:** Dalong Yang, Tongxiang Fan, Han Zhou, Jian Ding, Di Zhang

**Affiliations:** State Key Lab of Composites, Shanghai Jiaotong University, Shanghai, People's Republic of China; RMIT University, Australia

## Abstract

**Background:**

Rice husk, an agricultural bioresource, is utilized as a non-metallic bio-precursor to synthesize biogenic hierarchical TiO_2_/SiO_2_ (BH-TiO_2_/SiO_2_) and the products are applied to dye degradation.

**Methodology/Principal Findings:**

The as-prepared BH-TiO_2_/SiO_2_ samples are characterized by X-ray diffraction(XRD), X-ray photoelectron spectroscopy(XPS), nitrogen-adsorption measurement, UV-vis spectroscopy and electronic paramagnetic resonance (EPR). The results show that BH-TiO_2_/SiO_2_ possesses both anatase and rutile phases with amorphous SiO_2_ as background, which contains mesopore structure, and nitrogen derived from original rice husk is self-doped into the lattice. Besides, the light-harvesting within the visible-light range of BH-TiO_2_/SiO_2_ has been enhanced. Moreover, the catalytic activity of BH-TiO_2_/SiO_2_ has been proven by EPR, and both the photocatalytic activity and stability of BH-TiO_2_/SiO_2_ are improved as well, which has been illustrated by cycled degradation of methylene blue dye under irradiation.

**Conclusions/Significance:**

This work provides a good way to combine natural hierarchical porous structure with synthetic material chemistry based on available biomass in the vast natural environment for the sustainable development of human society, and extends potentials of biomass in applications such as photocatalysts, sunlight splitting water and so forth.

## Introduction

Bioresource and biochemical technology play a vital role in the sustainable development of human society [1]. Biomass is biological material derived from living organisms, which is a renewable resource and the primary source of energy for nearly half the world [2]. The traditional methods for utilizing biomass are mostly combusting for heat, which is common in developing countries, but harmful to global climate [3]. So we need to find new ways in utilizing biomass, instead of polluting the environment. On the other hand, millions of years of biological evolution evolved different kinds of special biological structures in nature that provides plenty of references for our scientific innovation. Nowadays, biomorphic mineralization has attracted a lot of attention, which is a technique that employs natural things as bio-templates for mineralization to synthesize bio-templated materials with morphologies and structures being similar to those of the bio-templates, which does well in utilizing biomass in the vast natural environment [4]. It inspires us that biomass could play a part in bio-precursors mineralization, because most biomass contains abundant non-metallic elements with hierarchical porous structure which covers the scale of micro, meso and macro. In conclusion, materials derived from biomass—biogenic materials, are combination of natural hierarchical porous structure with synthetic material chemistry, which could also contribute to the natural environment and play an important role in the field of biochemistry and bioresource engineering.

As a typical environment-friendly photocatalyst, Titanium dioxide (TiO_2_) has high efficient photoactivity as well as its stability with the low cost. It has been widely used in some important areas, such as water treatment, air purification, organic matter degradation, and so on [5]. However, the relatively large band gap allows TiO_2_ to absorb UV with wavelength less than 387 nm, which is assessed at only 5 per cent of total solar energy reaching the earth's surface. Currently, all kinds of efforts have been made to improve its light-harvesting and photocatalytic efficiency, especially within the visible-light range. One method is to fabricate TiO_2_ with various nanostructures such as nanoparticles, nanotubes or nanoporous frameworks [6], which inspired the effect of different nanostructure of materials could be helpful and made a landmark contribution to the research of materials. In recent years, there have been great interests in hierarchical porous structure, which can also enhance phtocatalytic activity of TiO_2_, because this hierarchically porous structure not only provides a convenient pore-wall system but also optimizes the transport of matter by minimizing the pressure drop over the material [7]. Another method of improving the light-harvesting properties of TiO_2_ within the visible-light range is to dope some elements as N [8], S and C or metallic nanoparticles as Au or Ag [9]. Among all element-doped method, N-doped method is considered to be effective as it could form a localized state slightly above the valence band in the band gap which could cause band narrowing, and it could also reduce the formation energy of oxygen vacancies [10]. There could be two types of N-doped: organic and inorganic. The former includes urea and other organic compounds [8]; the latter includes ammonia and other inorganic compounds [10]. These methods usually need to introduce additional source of nitrogen, which are complex and cumbersome. Moreover, mixed-oxide system containing TiO_2_ has attracted more and more attention in the field of photocatalyst, such as TiO_2_/SiO_2_, TiO_2_/Al_2_O_3_, TiO_2_/ZrO_2_ [11], [12], which has enhanced photocatalytic performance. Among all mixed-oxide system containing TiO_2_, titanium-silicon oxides have been found to enhance photocatalytic activity more efficiently, because silica (SiO_2_) has a good property in light-harvesting within visible range [11].

As agricultural bioresource, rice husk contains large mounts of silicon, so we try to develop a new method to synthesize titanium-silicon oxides by utilizing rice husk with calcination method [13], [14]. On the other hand, most biomass including rice husk contains abundant non-metallic elements such as C, H, O and N [15], which indicates that nitrogen in rice husk could be self-doped into mixed-oxide system during synthesis. Furthermore, the biological structure is hierarchical in nature [16], and rice husk is a typical example of which with large surface area and porosity [17], so the structure of that could contribute to adsorption. Such structural features of rice husk inspire us to replicate them on functional materials with special functional properties. Thus, we except to find a new way in utilizing rice husk effectively for inheriting its advantages of both chemical composition and microstructure. In addition, the products are expected to contribute to environmental protection.

In this study, we propose rice husk as non-metallic precursor to synthesize TiO_2_, because silicon is contained in rice husk, we expect it could be converted to SiO_2_ with calcination to synthesize mixed-oxide system as TiO_2_/SiO_2_. Besides, nitrogen in rice husk is expected to be self-doped into the mixed-oxide system during synthesis as well as the inheritance of its hierarchical porous structure, resulting in biogenic hierarchical TiO_2_/SiO_2_, hereafter refer to BH-TiO_2_/SiO_2_. The sol-gel coating is employed for the replication of the porous framework of the rice husk. The BH-TiO_2_/SiO_2_ samples are used for dye degradation, which is an example of pollutants degradation [18], [19]. This work is a combination of bioresource engineering and applied chemistry, and would develop a new method to protect the natural environment by utilizing useful biomass in the vast environment.

## Results

### Composition characterization of BH-TiO_2_/SiO_2_

**Figure 1:**
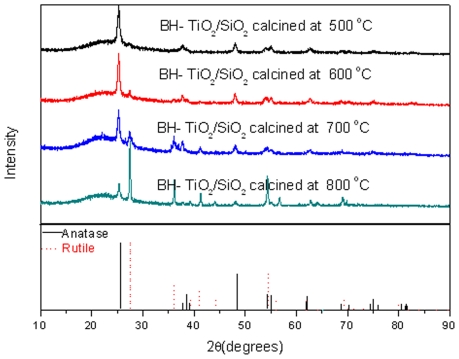
XRD patterns of BH-TiO_2_/SiO_2_.

XRD patterns of BH-TiO_2_/SiO_2_ are shown in Fig. 1 and patterns of all the stages of synthesis are shown in Fig. S1. The XRD diagrams of samples are very similar in Fig. S1. The peak position of the XRD diagrams was observed at 2θ = 22° in Fig. S1. The XRD diagrams of samples, shown in Fig. S1, indicate that samples are X-ray amorphous SiO_2_. [20] They didn't show TiO_2_ crystalline structure, such as anatase or rutile in Fig. 1. It shows clearly that all the BH-TiO_2_/SiO_2_ samples possess amorphous SiO_2_ phase as background in Fig. 1 [21], which are derived from the rice husk containing Si element. The difference between all the BH-TiO_2_/SiO_2_ samples is the proportion of anatase and rutile phase. As a result, we can infer that TiO_2_ was not generated before calcination, but obtained after calcination by comparing Fig. 1 with Fig. S1. The anatase phase shows a greater photocatalytic activity than the rutile phase, but the TiO_2_ powder with both anatase and rutile phases shows a much greater photocatalytic activity than the pure anatase powder or the pure rutile powder because of the effect of the rutile phase [22]. The average crystalline sizes of BH-TiO_2_/SiO_2_, calculated by Williamson-Hall method, are 29.2 nm, 30.7 nm, 36.8 nm and 45.8 nm for BH-TiO_2_/SiO_2_ from samples calcined at the temperature 500°C, 600°C, 700°C and 800°C, respectively. These grain sizes on the nanoscale may relate with high specific surfaces. We can still find the anatase phase from BH-TiO_2_/SiO_2_ calcined at 800°C, which suggests lattice effect of mixed-oxide system as TiO_2_/SiO_2_ [23].

**Figure 2:**
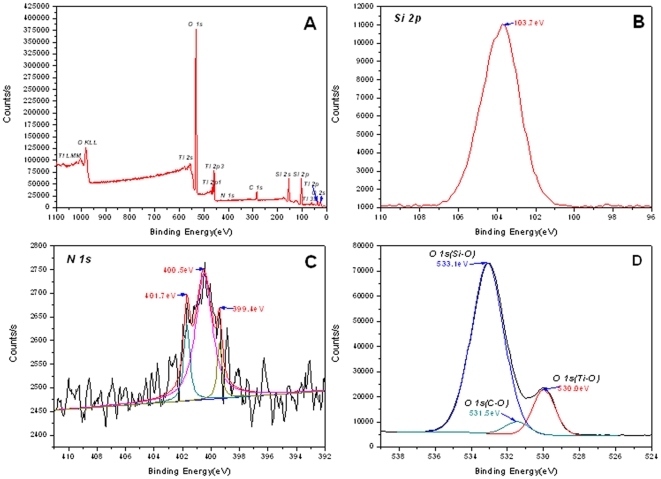
XPS patterns of BH-TiO_2_/SiO_2_ calcined at 500°C. (A) the whole survey; (B) high-resolution spectra of Si_2P_; (C) high-resolution spectra of N_1S_; (D) high-resolution spectra of O_1S_.

XPS tests identified the presence of doped N in TiO_2_/SiO_2_ network. The whole XPS survey of BH-TiO_2_/SiO_2_ calcined at 500°C (Fig. 2A) demonstrates Ti and Si exist in the oxides, it also demonstrates that N which is originated from natural rice husk exists in the oxides and that its atomic content is 0.55%. Mineral elements in rice husk, such as K, Ca, Na are not detected, implicating that the treatment with dilute HCl effectively got rid of them successfully. The high-resolution scanning of Si_2P_ displayed in Fig. 2B shows one peak at 103.7 eV [24], which demonstrates the existence of SiO_2_. The high-resolution scanning of N_1S_ displayed in Fig. 2C shows three peaks, at 399.4 eV, 400.5eV and 401.7 eV. The N_1S_ peak at 399.4 eV is attributed to N anion in O-Ti-N or O-Si-N bonds, where the oxygen sites in O-Ti-O or O-Si-O bonds are partly substituted by nitrogen atoms [24], [25], which is helpful for photocatalytic within the visible range. The latter two peaks are at 400.5 eV and 401.7 eV, which indicate that the N is assigned to molecularly chemisorbed nitrogen species [26], [27]. On the other hand, the red-shifts of BH-TiO_2_/SiO_2_ at the edge of the UV and visible light in the following light-harvesting experiments could also prove the effect of N-doped phenomenon. The high-resolution scanning of O_1S_ displayed in Fig. 2D shows that the broad O_1S_ region can be fitted by three peaks, as follows, Ti-O bonds [28], Si-O bonds [29] and C-O bonds [30]. We could find only one peak at about 285 eV from the high-resolution scanning of C_1S_ in Fig. S2, which excludes the possibility of C-doped [31]. The XPS tests demonstrate that N element is self-doped into BH-TiO_2_/SiO_2_ from original rice husk during synthesis, which suggests an effective way of enhancing the content of the doping N for these oxides derived from biomass.

### Structure characterization of BH-TiO_2_/SiO_2_

**Figure 3:**
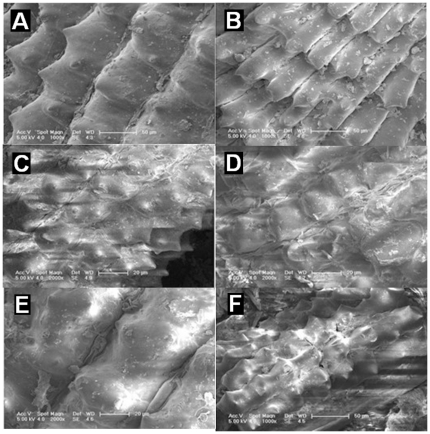
FE-SEM images of the surface structure. (A) original rice husk; (B) rice husk calcined at 500°C; (C) BH-TiO_2_/SiO_2_ calcined at 500°C; (D) BH-TiO_2_/SiO_2_ calcined at 600°C; (E) BH-TiO_2_/SiO_2_ calcined at 700°C; (F) BH-TiO_2_/SiO_2_ calcined at 800°C.

As it has been known that most biomass has hierarchical pore structure [16], we expect that hierarchical pore structure of rice husk could be replicated to BH-TiO_2_/SiO_2_. The hierarchical structure need to be characterized by different means. Our group has demonstrated that our synthesis method could successfully replicate the hierarchical pore structure of biomass [32], [33]. We could find that the morphology of the samples has not been seriously destroyed with calcination method from Fig. 3. The hierarchical structure covers both the micron and nano scales. The micron pores which could be proven by SEM are shown in Fig. S3.

**Figure 4:**
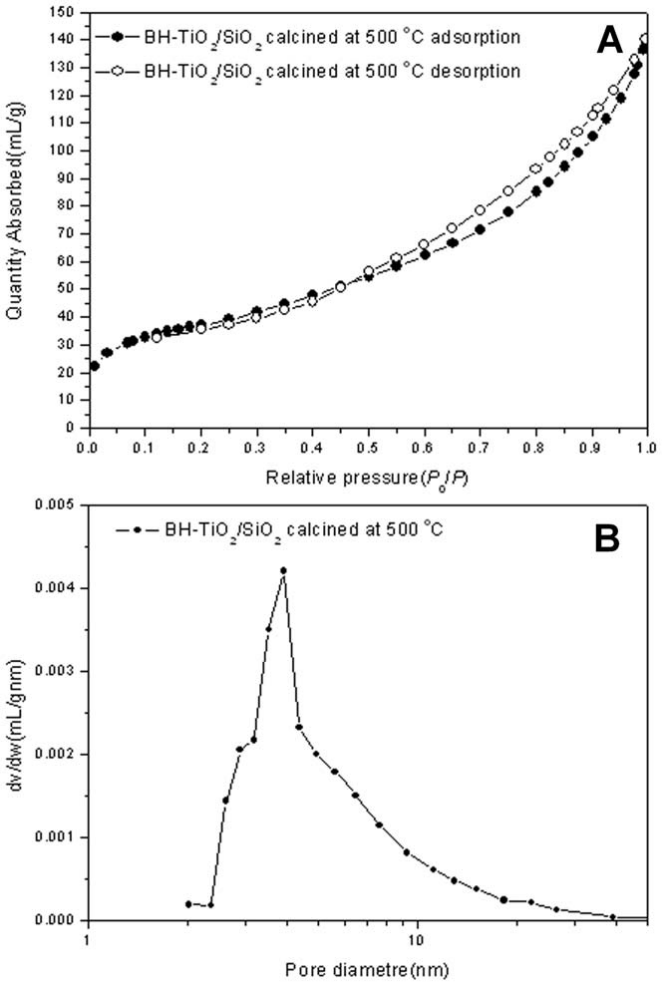
Nitrogen adsorption results of BH-TiO_2_/SiO_2_ calcined at 500°C. (A) adsorption-desorption isotherms; (B) pore-size distribution curves.

Now, the nitrogen-adsorption measurements have been used to characterize the nano-scale porous structures of the BH-TiO_2_/SiO_2_ samples. Fig. 4A and B displays the adsorption-desorption isotherms and poresize-distribution curves respectively of BH-TiO_2_/SiO_2_ calcined at 500°C. The other kinds of BH-TiO_2_/SiO_2_ samples exhibit similar isotherms. According to the IUPAC classification, this is a H3-type type-IV isotherm [34]. From Fig. 4B, we can see that the pore sizes on the nanoscale for the BH-TiO_2_/SiO_2_ calcined at 500°C are distributed between 2 nm and 40 nm, centered on 4 nm, the average pore width is about 6.9 nm. Besides, the BET surface area is about 131.9 m^2^g^−1^. The mesopore structure of the BH-TiO_2_/SiO_2_ in a wide range could offer more adsorption, which is helpful for the photocatalytic reaction. Besides, there could be some phenomenon of the enrichment of material to be degraded, which could speed up the reaction of degradation. In addition, the high surface area is helpful for light-harvesting, which also offers large absorption area. Meanwhile, the hierarchical pore structure of rice husk has been successfully replicated, which could be helpful for electrons transferring from one electron-hole to another. As a result, the structure of the BH-TiO_2_/SiO_2_ is potentially helpful for improving the efficiency of light-harvesting and photocatalytic activity.

### Light-harvesting properties of BH-TiO_2_/SiO_2_

**Figure 5:**
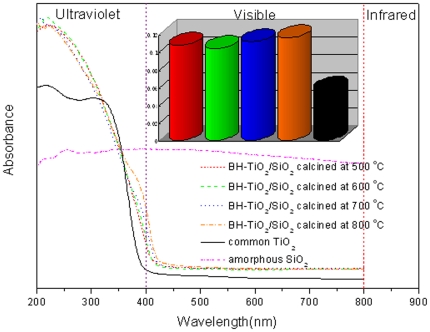
UV-vis absorption spectra of BH-TiO_2_/SiO_2_, common TiO_2_ and amorphous SiO_2_. (with a bar graph of the average absorbance intensities within the visible range for BH-TiO_2_/SiO_2_ and common TiO_2_).

The light-harvesting properties of the BH-TiO_2_/SiO_2_ are characterized by UV-vis spectroscopy. Figure 5 displays the UV-vis absorbance of the BH-TiO_2_/SiO_2,_ common TiO_2_ and amorphous SiO_2_. Compared with common TiO_2_, BH-TiO_2_/SiO_2_ shows two prominent features. The first is that the overall visible-light absorbance intensities are enhanced. The second is that the band-gap-absorption onsets, at the edge of the UV and visible light, show a clearly red-shift. The average absorbance intensities within the visible range (400 nm–800 nm) increase by 87%, 80%, 94%, 102% for the BH-TiO_2_/SiO_2_ calcined at 500°C, the BH-TiO_2_/SiO_2_ calcined at 600°C, the BH-TiO_2_/SiO_2_ calcined at 700°C and the BH-TiO_2_/SiO_2_ calcined at 800°C, respectively. Although the BH-TiO_2_/SiO_2_ calcined at 600°C has the highest absorbance in the ultraviolet light range, the BH-TiO_2_/SiO_2_ calcined at 800°C has the highest absorbance in the visible light range. The existence of amorphous SiO_2_ is helpful for light-harvesting within the visible range, which could explain the reason why BH-TiO_2_/SiO_2_ has a higher light-harvesting than common TiO_2_ within the visible range. On the other hand, the band-gap-absorption onsets, at the edge of the UV and visible light, show red-shifts of about 25 nm for the BH-TiO_2_/SiO_2_ calcined at 500°C, the BH-TiO_2_/SiO_2_ calcined at 600°C, the BH-TiO_2_/SiO_2_ calcined at 700°C, and 40 nm for the BH-TiO_2_/SiO_2_ calcined at 800°C. This is caused by the doping of N, which forms a localized state beyond the valence band [35]. The results suggest that there are more photo-generated electrons in the BH-TiO_2_/SiO_2_ than common TiO_2_, as a result, the efficiency of photocatalytic could be higher. The BH-TiO_2_/SiO_2_ realizes the synergy of both the structure-introduced enhancement of the visible-light harvesting and the red-shift of the band-gap-absorption onsets induced by self-doping of nitrogen. The results also prove that the employed procedure could be successful in both the introduction of the templates structural features into the new materials and the sensitization towards visible light by N-doping simultaneously in only one step. On the other hand, the hierarchical pore structure in a wide range could also be helpful for light-harvesting.

### Photocatalytic properties of BH-TiO_2_/SiO_2_

**Figure 6:**
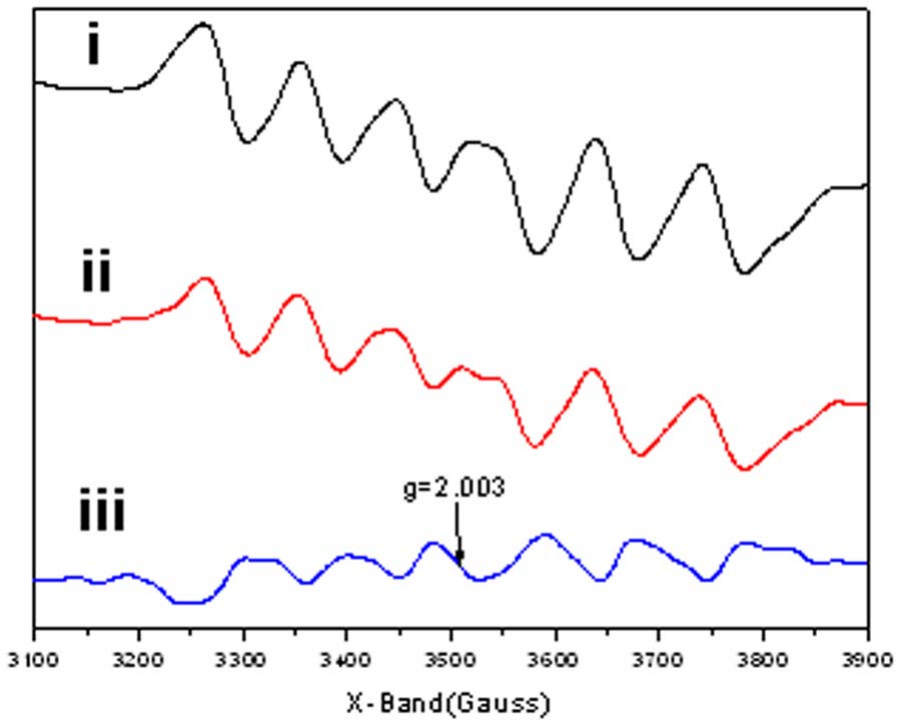
EPR spectra of BH-TiO_2_/SiO_2_ calcined at 500°C under 300K. i) before irradiation; ii) after irradiation for 30 mins; iii) result of subtraction of (i) from (ii).(All data of EPR spectra has been smoothed.)

In order to demonstrate the catalytic activity of the BH-TiO_2_/SiO_2_, electronic paramagnetic resonance (EPR) studies are performed. The EPR patterns (i) and (ii) in Figure 6, corresponding to BH-TiO_2_/SiO_2_ before and after irradiation for 30 mins, show some clearly signals, which means the number of unpaired electron-hole in oxidation states[36]. Meanwhile, there have been much electrons transferring from one electron-hole to another, which is useful for photocatalytic [37]. Especially, we could see the new narrow signals with *g* = 2.003, which could be ascribed to N-O species in TiO_2_ lattice[38]. It is well known trapped electrons could reduce Ti^4+^ to form Ti^3+^ paramagnetic species according to the following process: Ti^4+^ + e^−^ = Ti^3+^ . In addition, the resonances at g values in the range of 2.0 to 2.08 are known to be due to photogenerated holes trapped by subsurface lattice oxygens[39]. These holes, localized on the oxygen vacancies, react with the O_2_^−^ and OH^−^ to form O and OH radicals on the catalyst surface, both of which are reactive species[40]. The EPR results prove that the life time of the photogenerated holes and electrons in BH-TiO_2_/SiO_2_ is long enough, which reflects the higher charge-separation efficiency of BH-TiO_2_/SiO_2_, and this could lead to higher photocatalytic activity[41], [42].

**Figure 7:**
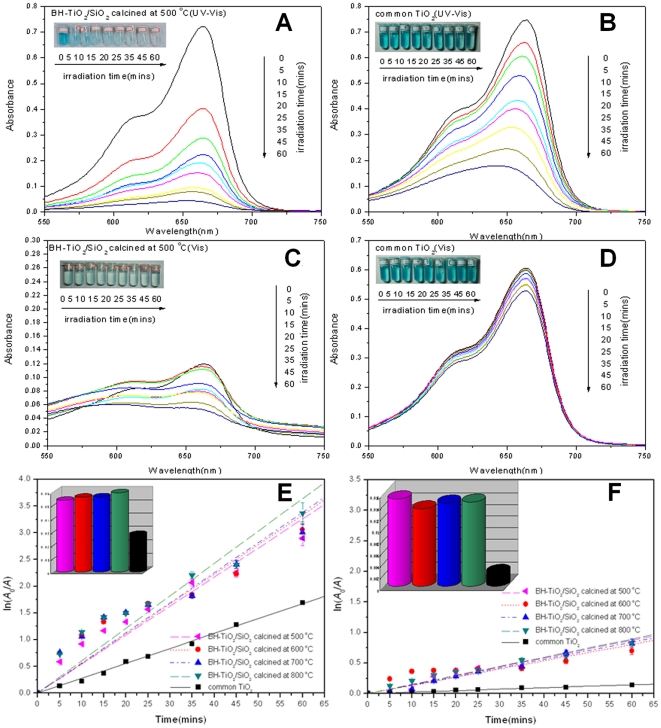
Photocatalytic degradation studies of BH-TiO_2_/SiO_2_ and common TiO_2_ under UV and visible light. (A) Changes in the UV-vis absorption spectra of methylene blue as a function of irradiation time in the presence of BH-TiO_2_/SiO_2_ calcined at 500°C under UV and visible light. (B) Changes in the UV-vis absorption spectra of methylene blue as a function of irradiation time in the presence of common TiO_2_ under UV and visible light. (C) Changes in the UV-vis absorption spectra of methylene blue as a function of irradiation time in the presence of BH-TiO_2_/SiO_2_ calcined at 500°C under visible light. (D) Changes in the UV-vis absorption spectra of methylene blue as a function of irradiation time in the presence of common TiO_2_ under visible light. (Insets in a, b, c and d are corresponding color variations over irradiation time.) (E) Kinetic study of degradation of methylene blue solution in the presence of BH-TiO_2_/SiO_2_ and common TiO_2_ under Xe lamp irradiation, with a bar graph of the degradation rates in the UV and visible range. (F) Kinetic study of degradation of methylene blue solution in the presence of BH-TiO_2_/SiO_2_ and common TiO_2_ under Xe lamp irradiation, with a bar graph of the degradation rates in the visible range. (Cut-off filters at 400 nm are employed to remove wave lengths shorter than 400 nm to get the desired visible light).

Photocatalytic studies are carried out by studying the decomposition reaction of methylene blue dye in the presence of BH-TiO_2_/SiO_2_ and common TiO_2_ under UV and visible-light (400 nm) irradiation. The material used in this study is in the form of hierarchical porous particles. The degradation process obeys pseudo-first-order kinetics and the degradation rate could be obtained by plotting the natural logarithm of the absorbance against irradiation time. In order to correct accident experimental errors, error bars are introduced to graphs by calculating each data's standard deviation. We can easily find that the characteristic peak of methylene blue is at 665 nm in Fig. 7A, B, C and D. After 1 hour adsorption equilibrium, we can find the initial characteristic peak of methylene blue decreased different from each other, which indicate the adsorption effect of each sample. After 1 hour adsorption equilibrium, we could find that the decrease of initial characteristic peaks of methylene blue is more obvious in Fig. 7C compared to Fig. 7A owing to more BH-TiO_2_/SiO_2_ samples. So we could prove the effective adsorption of BH-TiO_2_/SiO_2_ samples. However, as the case of common TiO_2_ samples in Fig. 7B and D, after 1 hour adsorption equilibrium there was no obvious change of initial characteristic peaks of methylene blue. As a result, BH-TiO_2_/SiO_2_ has a stronger adsorption effect than common TiO_2_, which suggest its potentials in high photocatalytic activity. We can also find that the characteristic peak of methylene blue decreased gradually and disappeared after irradiation for 60 minutes in the presence of BH-TiO_2_/SiO_2_ calcined at 500°C in Fig. 7A and C, which indicates that methylene blue was completely or partly degraded by BH-TiO_2_/SiO_2_ calcined at 500°C. For comparison in Fig. 7B and D, methylene blue was degraded a little after the same time of irradiation in the presence of common TiO_2_. In order to evaluate the degradation rates quantitatively, we introduce pseudo-first-order kinetics to process the absorbance data during the degradation. The degradation rate can be calculated by fitting curves in Fig. 7E and F. The degradation rate of BH-TiO_2_/SiO_2_ calcined at 500°C specimens under UV and visible-light is 0.0532 min^−1^, almost twice of that of common TiO_2_, which is 0.0280 min^−1^ under UV and visible-light. At the same time, the degradation rate of BH-TiO_2_/SiO_2_ calcined at 500°C specimens only under visible-light is 0.0149 min^−1^, six times more than that of common TiO_2_, which is 0.0024 min^−1^ only under visible-light. Moreover, the average degradation rate of BH-TiO_2_/SiO_2_ is 0.0559 min^−1^ under UV and visible-light, and 0.0143 min^−1^ only under visible-light. As a result, the dye enrichment which is enhanced by hierarchical pore structure can speed up the degradation reaction. Meanwhile, we can see it clearly that the photocatalytic efficiency of BH-TiO_2_/SiO_2_ is much higher than that of common TiO_2_, not only within the UV range, but also within the visible range. Especilly, BH-TiO_2_/SiO_2_ shows good photocatalytic properties within the visible range, which suggests an outlook of implication in optical properties in future. On the other hand, other dyes, such as congo red, methyl orange, rhodamine 6G and malachite green, can also be degraded by BH-TiO_2_/SiO_2_ under UV and visible-light (400 nm).

**Figure 8:**
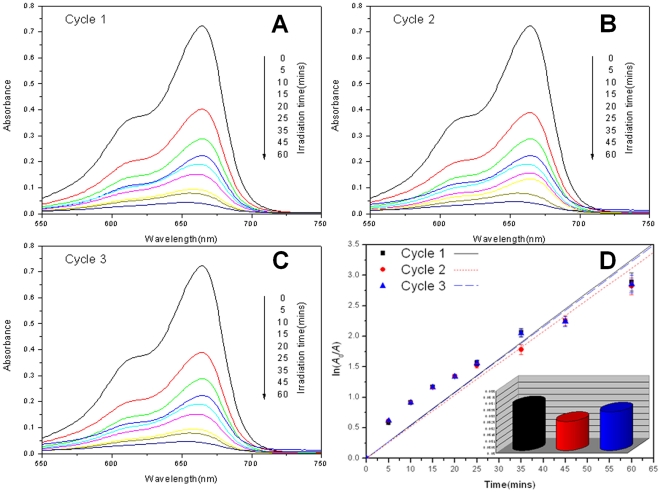
Cycled photocatalytic degradation studies of BH-TiO_2_/SiO_2_ calcined at 500°C under Xe lamp. (A) Cycle 1; (B) Cycle 2; (C) Cycle 3; (D) Kinetic study of cycled degradation.(with a bar graph of the degradation rates for each cycle).

The results of cycled photocatalytic degradation of methylene blue are shown in Fig. 8. Error bars are also introduced to correct accident experimental errors. The degradation rates of cycle 1, 2 and 3 are 0.0531 min^−1^, 0.0518 min^−1^ and 0.0526 min^−1^. It is almost the same degradation rates. As a result, the repeated use of BH-TiO_2_/SiO_2_ doesn't reduce its photocatalytic activity, which demonstrates BH-TiO_2_/SiO_2_ has good photocatalytic stability.

Compared with common TiO_2_, the BH-TiO_2_/SiO_2_ are endowed with great photocatalytic activity, according to the UV-vis absorption spectra, under UV and visible-light (400 nm) irradiation. This is not only the advantage of their structures, but also the advantage of their components. The hierarchical porous structure and the high BET surface area are both helpful for absorption and reaction sites for the photocatalytic reaction. On the other hand, Si is successfully introduced as an important part of TiO_2_/SiO_2_ with great photocatalytic property. The successful self-doping of N can expand the wavelength absorption range and enhance the light-harvesting within the visible range, which is helpful for photocatalytic activity within the visible range.

## Discussion

This work employed rice husk as prototype to demonstrate its potential application in synthesizing hierarchical TiO_2_/SiO_2_. Compared with common TiO_2_, both the light-harvesting and photocatalytic properties of BH-TiO_2_/SiO_2_ are much enhanced. The method of serving for natural environment by drawing on the environment itself is far-reaching. Besides BH-TiO_2_/SiO_2_, this method may also be applied to synthesize other element-self-doped TiO_2_ in only one step, such as kelp which contains iodine, loofah which contains phosphorus. This work is of great meaning in combining biological engineering and biochemistry, providing a good way combining natural hierarchical porous structure with synthetic material chemistry and extending potentials of biomass in applications such as photocatalysts, sunlight water splitting and so forth.

## Materials and Methods

### Synthesis procedure

Synthesis Method: The natural rice husk was treated with 5% HCl solution under vacuum at 10°C for 24 h to get rid of K, Ca and Na irons, with the color of rice husk turning from yellow to deep-yellow. After being rinsed with pure water for 24 h, the as-treated rice husk was treated with pure ethanol (EtOH) solution under vacuum at 10°C for 24 h to provide large mounts of functional groups such as hydroxyls. After being rinsed with pure water for 24 h again, the as-treated rice husk was stressed with 10% TiCl_3_ solution under vacuum at 10°C for 120 h, since the existence of many functional groups such as hydroxyls, Ti ions were easily introduced into the rice husk. The stressed rice husk then underwent a graded dehydration process with 30%, 50%, 70%, 95% and 100% EtOH successively, each concentration for half an hour. A 3% tetrabutyl titanate (Ti(OBu)_4_)/EtOH solution accompanied with acetylacetone (acac) (Ti(OBu)_4_:EtOH:acac–v:v:v = 3:100:0.1) was employed as the precursor for sol-gel coating. (Ti(OBu)_4_ was used to protect samples against deformation. Acac was used to control the hydrolysis of the precursor. The dehydrated samples were infiltrated in the solution under vacuum for 48 h at 10°C, rinsed with EtOH and then left in air for hydrolysis by moisture for 2 h at 10°C. Afterwards, the samples were desiccated in an aerated oven at 25°C, 60°C and 105°C successively, with each temperature for 2 hours. Then, the samples were calcined in air at 280°C for 2 h, with a ramping rate of 1°C min^−1^. At last, the as-treated samples were clacined at 500°C, 600°C, 700°C, 800°C for 2 h, respectively, with a ramping rate of 1°C min^−1^_._ For comparison, common TiO_2_ was synthesized by common sol-gel method [43] under the same conditions with BH-TiO_2_/SiO_2_.

### Characterization

The crystal phase of the BH-TiO_2_/SiO_2_ samples was examined by X-ray diffraction (XRD) on a Bruker-AXS X-ray diffractometer system with Cu Kα radiation at 40 kV and 100 mA. The spectra were recorded in the 2*θ* range from 10° to 90° with a scanning step of 0.02°/s. The average crystalline sizes were calculated by Williamson-Hall method, expressed as follows: *β_i_(cosθ_i_)/λ = K/D+4ε(sinθ_i_)/λ*, where *β_i_* is the integral breadth (in radius 2*θ*) of the *i*th Bragg reflection peak positioned at 2*θ_i_*; K is a constant with the value of 0.89; *λ* = 0.154 nm is the wavelength of the X-rays; *D* is the average size of the crystallites and *ε* is the microstrain, whose distribution is isotropic. *D* and *ε* were calculated through a least-squares fit.

The presence and states of Ti, Si and N in BH-TiO_2_/SiO_2_ were identified by XPS, performed on a Thermo ESCALAB 250 spectrometer with monochromatized Al Kα X-ray (hν = 1486.6 eV) at a pass energy of 20eV. All the binding energies were calibrated by using the contaminant carbon (C_1S_ = 284.8 eV) as a reference.

Cross sections of the 10 mm samples were also collected on conductive glass slides, together with BH-TiO_2_/SiO_2_ derived from rice husk. They were sputtered with gold and observed using field-emission scanning electron microscopy (FESEM) (FEI SIRION 200) operated at 5 kV.

The surface areas and pore-size distributions were evaluated by nitrogen-adsorption measurements, operated at 77 K on a Micromeritics ASAP 2020 adsorption analyzer. All of the BH-TiO_2_/SiO_2_ samples were degassed at 200°C and 10^−6^ Torr for 5 h prior to the measurements. The pore-sizes and distribution curves were derived from the adsorption isotherm by employing the Barrett-Joyner-Halenda (BJH) method, and the surface areas were calculated through the Brunauer–Emmett–Teller (BET) equation.

UV-vis absorption spectroscopy of all of the samples was recorded using a Varian Cary UV-vis-NIR spectrophotometer in the spectral range 200–800 nm. 0.25 g of each sample was pressed between two pieces of quartz glass within the 3×3 cm area to cover the aperture through which the excitation light passed. A BaSiO_4_ plate was used as the basic line for the spectra. The same quality for the all of the BH-TiO_2_/SiO_2_ samples was taken during the measurement.

EPR measurements were carried out on a Bruker EMX-8/2.7 X-band EPR spectrometer operating in the X-band at 9.875 GHz and 19.870 mW. Portions of the solid samples (about 20 mg) were introduced into a spectroscopic quartz probe cell and the measurements were taken at the room temperature (300 K) by placing the cell in a quartz flask filled with room air. For irradiation of the samples, the probe cells loaded with samples were irradiated using a high-pressure mercury lamp with a maximum wavelength of 365 nm. Following illumination for 30 min, the cell was immediately transferred to the spectrometer cavity for EPR analysis.

### Measurement of photocatalytic activity and stability

To evaluate the photocatalytic activities of BH-TiO_2_/SiO_2_, degradation experiments of methylene blue nonahydrate (C_16_H_18_C_l_N_3_S•3H_2_O) in aqueous solution was carried out in the presence of BH-TiO_2_/SiO_2_ and common TiO_2_ as follows. 40 mg for degradation under UV and visible light, 100 mg for degradation only under visible light corresponding TiO_2_ sample were dispersed in 100 mL of 10^−5^ mol/L methylene blue solution, respectively. The TiO_2_ aqueous suspension was stirred in dark for 1 hour to reach its adsorption equilibrium and then it was irradiated under a 500 W Xe lamp, whose emission spectrum was the nearest to the solar spectrum and which was thus the most widely used light source of solar simulator. Cut-off filters at 400 nm were employed to decide whether to remove wave lengths shorter than 400 nm to get the desired visible light or not. Degradation was monitored by taking sample solutions at 0, 5, 10, 15, 20, 25, 35, 45, 60 minutes respectively during the irradiation. These sample solutions were centrifuged with 3000 r/min for 5 minutes. Then, the supernatants were tested in sequence using a 25 Lamda UV-Vis spectrometer to get the absorption spectra. The rate of degradation was assumed to obey pseudo-first-order kinetics and the degradation rate constant, *k*, was obtained according to the following equation: *ln(A_0_/A) = kt*, where *A_0_* is the initial absorbance, namely, the characteristic absorbance peak of original methylene blue; *A* is the characteristic absorbance peak of methylene blue after a *t* time degradation.

To evaluate the photocatalytic stability of BH-TiO_2_/SiO_2_, three circles of methylene blue photocatalytic degradation were carried out in the presence of BH-TiO_2_/SiO_2_ calcined at 500°C without any cut-off filters to get the desired UV and visible light. The circled degradation was monitored by taking sample solutions at 0, 5, 10, 15, 20, 25, 35, 45, 60 minutes respectively during the photocatalytic reaction. After each degradation circle, the degraded solution with BH-TiO_2_/SiO_2_ photocatalyst suspended in it was centrifuged with 3000 r/min for 5 minutes. Then the supernatant was drawn out with a dropper and pure water was added to the remaining precipitate for washing. The suspension of BH-TiO_2_/SiO_2_ and pure water was centrifuged to get the clean BH-TiO_2_/SiO_2_ photocatalyst. The obtained wet BH-TiO_2_/SiO_2_ was dried at 60°C for the next degradation circle.

## Supporting Information

Figure S1 **XRD patterns of all the stages of synthesis.** (A) Natural rice husk. (B) After incubation of TiCl_3_. (C) Before heat treatment.
(TIF)

Figure S2 **XPS patterns of BH-TiO_2_/SiO_2_ calcined at 500°C: high-resolution spectra of C_1S_.**
(TIF)

Figure S3 **FE-SEM images of the hierarchical pore structure in micron scale.**
(TIF)
