# Supplementary figures and images for: Biogenic Hierarchical TiO2/SiO2 Derived from Rice Husk and Enhanced Photocatalytic Properties for Dye Degradation
Source: PLoS One. 2011 Sep 9;6(9):e24788. doi: 10.1371/journal.pone.0024788 (PMC3170388; doi:10.1371/journal.pone.0024788)

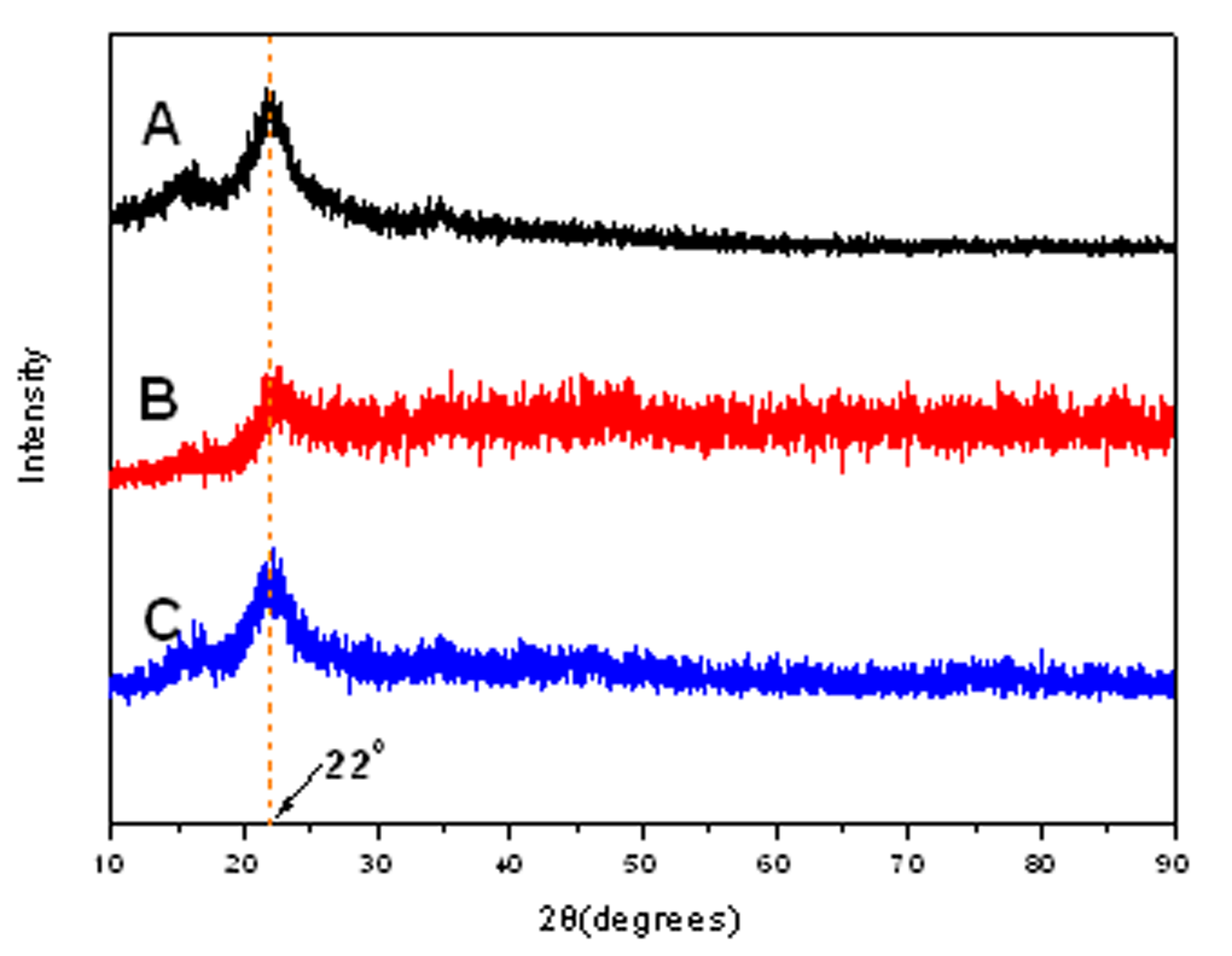

Supplement: Figure S1 — XRD patterns of all the stages of synthesis. (A) Natural rice husk. (B) After incubation of TiCl3. (C) Before heat treatment. (TIF) [file pone.0024788.s001.tif]

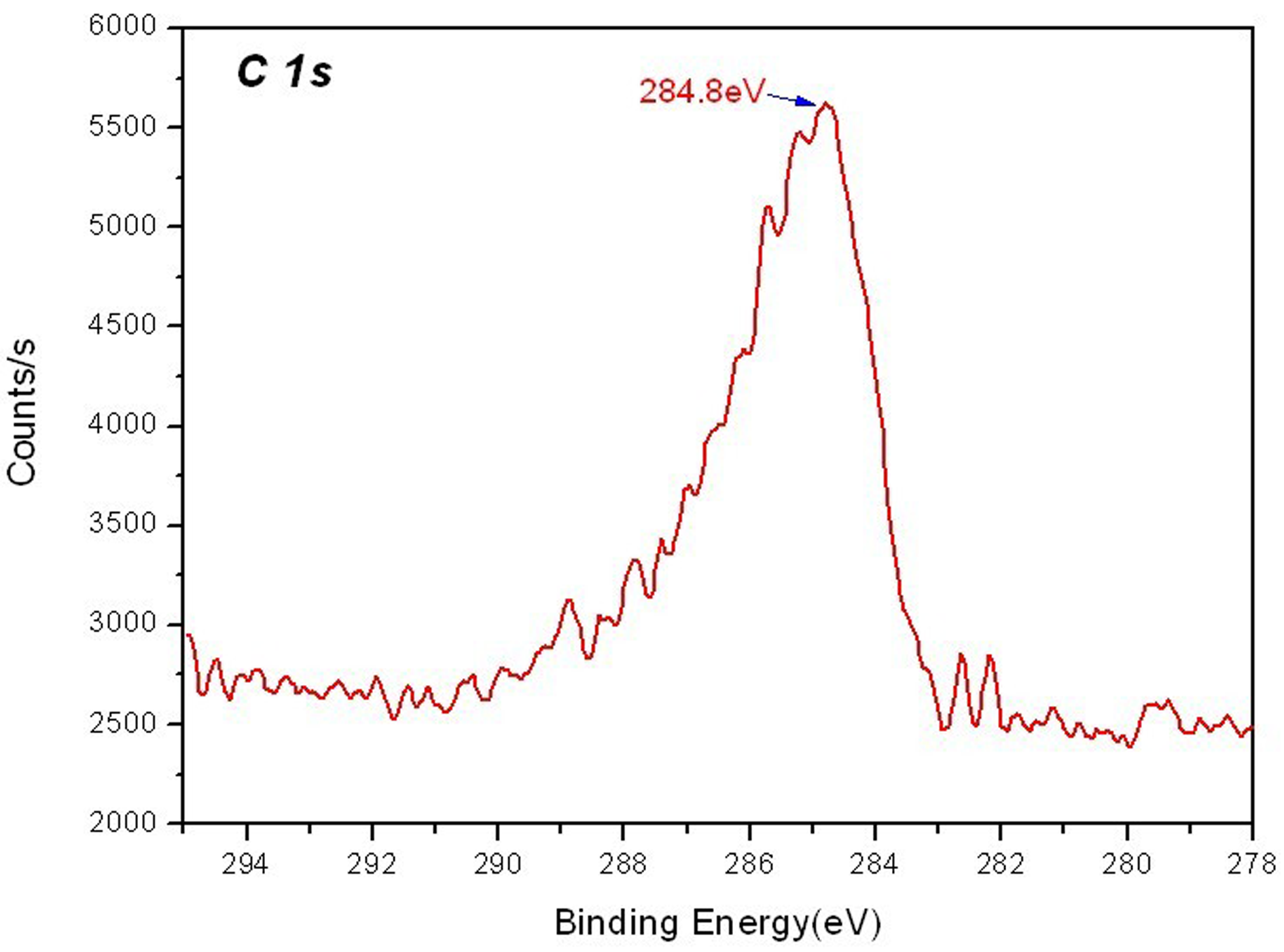

Supplement: Figure S2 — XPS patterns of BH-TiO2/SiO2 calcined at 500°C: high-resolution spectra of C1S. (TIF) [file pone.0024788.s002.tif]

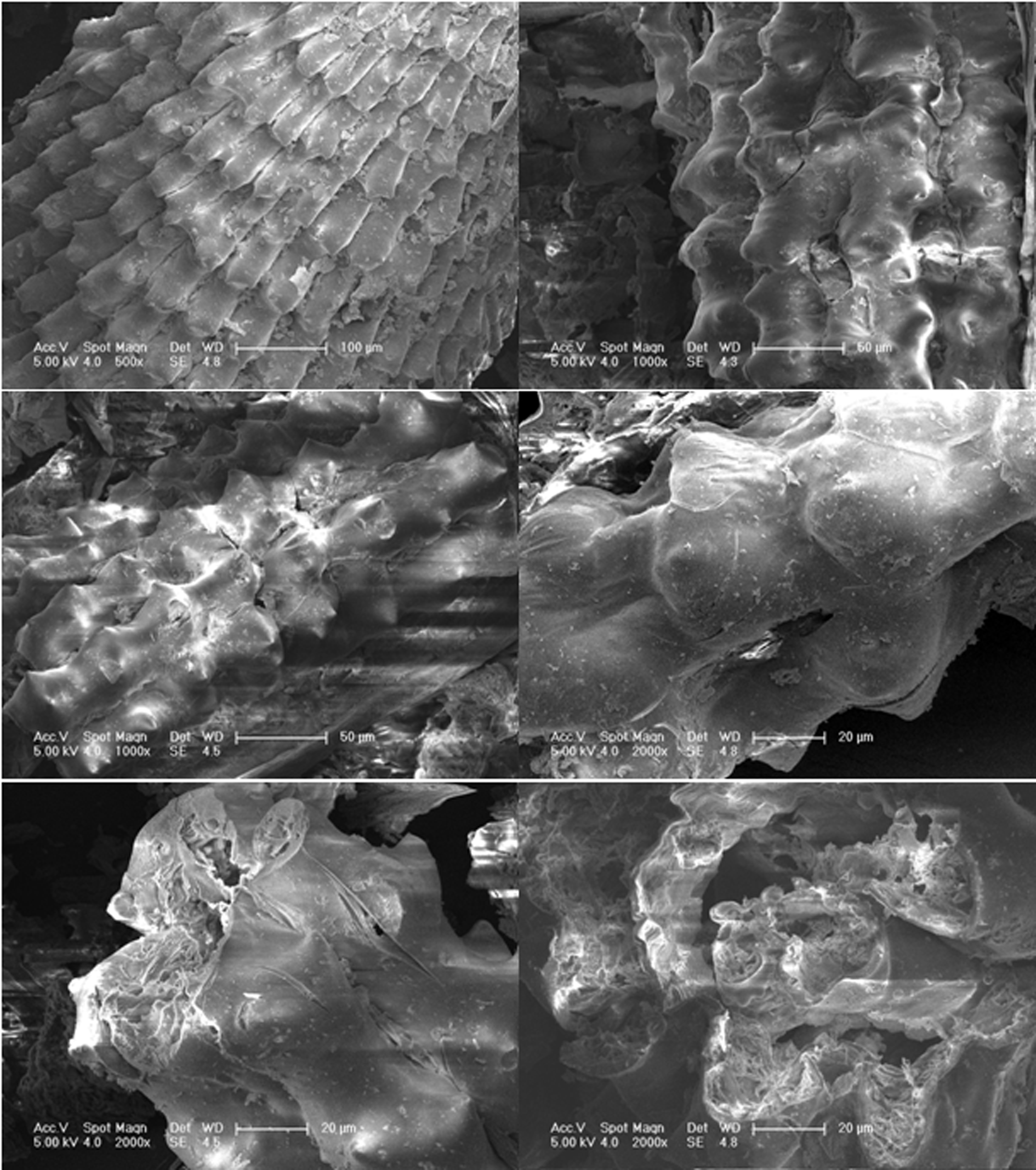

Supplement: Figure S3 — FE-SEM images of the hierarchical pore structure in micron scale. (TIF) [file pone.0024788.s003.tif]
